# Supplementary material for: Differences in the Inflammatory Response of White Adipose Tissue and Adipose-Derived Stem Cells
Source: Int J Mol Sci. 2020 Feb 6;21(3):1086. doi: 10.3390/ijms21031086 (PMC7037886; doi:10.3390/ijms21031086)
Supplement: Supplementary file 1 [file ijms-21-01086-s001.zip › ijms-703817_R1_Tab.S4.docx]

Tab.S4: Patient information. BMI: body mass index.

| **Donor-ID** | **sex** | **Age [year]** | **harvesting site** | **BMI** | **surgery type** |
| --- | --- | --- | --- | --- | --- |
| Donor 1 | female | 56 | abdomen | 24,7 | aesthetic |
| Donor 2 | female | 76 | thighs | 35,5 | aesthetic |
| Donor 3 | male | 25 | abdomen | 23,2 | aesthetic |
| Donor 4 | female | 26 | thighs | 21,0 | aesthetic |
| Donor 5 | male | 38 | abdomen | 27,8 | aesthetic |
